# Phenotypic profiling of ABC transporter coding genes in *Myxococcus xanthus*

**DOI:** 10.3389/fmicb.2014.00352

**Published:** 2014-07-18

**Authors:** Jinyuan Yan, Michael D. Bradley, Jannice Friedman, Roy D. Welch

**Affiliations:** Department of Biology, Syracuse UniversitySyracuse, NY, USA

**Keywords:** multicellularity, evolution, *Myxococcus xanthus*, biofilm, fitness, phenome

## Abstract

Information about a gene sometimes can be deduced by examining the impact of its mutation on phenotype. However, the genome-scale utility of the method is limited because, for nearly all model organisms, the majority of mutations result in little or no observable phenotypic impact. The cause of this is often attributed to robustness or redundancy within the genome, but that is only one plausible hypothesis. We examined a standard set of phenotypic traits, and applied statistical methods commonly used in the study of natural variants to an engineered mutant strain collection representing disruptions in 180 of the 192 ABC transporters within the bacterium *Myxococcus xanthus.* These strains display continuous variation in their phenotypic distributions, with a small number of “outlier” strains at both phenotypic extremes, and the majority within a confidence interval about the mean that always includes wild type. Correlation analysis reveals substantial pleiotropy, indicating that the traits do not represent independent variables. The traits measured in this study co-cluster with expression profiles, thereby demonstrating that these changes in phenotype correspond to changes at the molecular level, and therefore can be indirectly connected to changes in the genome. However, the continuous distributions, the pleiotropy, and the placement of wild type always within the confidence interval all indicate that this standard set of *M. xanthus* phenotypic assays is measuring a narrow range of partially overlapping traits that do not directly reflect fitness. This is likely a significant cause of the observed small phenotypic impact from mutation, and is unrelated to robustness and redundancy.

## Introduction

An organism's phenotype is considered to be the product of its genotype and environment. To the extent that this statement is true, then in the laboratory, where environment can be reproduced, genotype and phenotype should be more directly correlated. The problems involved in determining the exact nature of this correlation have been part of biological research for more than 100 years, and at present are collectively referred to as the genotype-to-phenotype problem (Benfey and Mitchell-Olds, 2008).

In most genotype-to-phenotype studies of a model organism, a strain that is considered “wild type” is genetically manipulated to produce mutant strains that can then be compared to wild type using assays that measure phenotypic traits. The wild type strain, although it has almost certainly evolved from its naturally occurring ancestor while in the laboratory, is assumed to be stable for the purposes of the study, and can therefore be used as a baseline for both genotype and phenotype. Large scale studies involving hundreds or thousands of mutant strains have now been reported for several model plant, animal and microbial systems.

A similar method that predates the term genotype-to-phenotype, is screening for a mutant phenotype. Traditional screens have been performed on almost every model organism, most of them multiple times, and they have succeeded in identifying and characterizing genetic elements that are involved in many biological processes. However, screens are typically designed to identify mutant strains that display extremely defective versions of one or a few mutant phenotypic traits, and this usually represents a very small percentage of the total population. For example, one study that used a gene deletion library covering 96% of open reading frames (ORFs) in *S. cerevisiae* found that only ~15% displayed slow growth on rich medium (Giaever et al., 2002), and a second study that used a genome-wide RNA interference (RNAi) screen in *D. melanogaster* found that only 438 out of 19,470 (<3%) of dsRNAs significantly reduced cell growth and viability (Boutros et al., 2004). This small percentage of discernible mutant phenotypes does not necessarily increase when more than one characteristic is measured; in an RNAi study in *C. elegans*, only 10% of genes were identified as exhibiting an impact on phenotypes that included three traits: development, growth and morphology (Kamath et al., 2003).

The majority of microbial genotype-to-phenotype studies focus on a single characteristic, growth rate (Liu et al., 2010; North et al., 2012), but a few have attempted to characterize more complex multicellular traits. In *Pseudomonas aeruginosa*, confocal microscopy was used to study biofilm density patterns in mutant strains using a high-throughput assay (Pommerenke et al., 2010), and in *Dictyostelium discoideum*, time lapse images of ~2000 mutant strains were used to identify and characterize changes in the formation of fruiting bodies (Sawai et al., 2007). In both of these studies, despite the acquisition of detailed image data, the percentage of strains displaying discernably mutant traits remained below 10%.

*Myxococcus xanthus* is a bacterium with a large but structurally simple genome (one 9 Mbp closed circle, no plasmids, 7355 predicted ORFs), and a complex multicellular phenotype. In the laboratory, on one of two different kinds of agar, a population of *M. xanthus* will either move and grow as a biofilm called a swarm, or undergo a developmental process that results in the formation of quiescent fruiting bodies. The environmental trigger that divides swarming and development is nutrient stress: feeding cells swarm and starving cells develop. Both processes exhibit coordinated cell movement, multicellular self-organization and, at least for development, cell differentiation (sporulation). More than one transposon mutagenesis screen has shown that ~0.5–1% of strains exhibit a loss-of-function in swarm motility or development (Kroos et al., 1986; Youderian et al., 2003; Youderian and Hartzell, 2006) and, given these reproducible small percentages, it would seem logical to conclude that more screening is unlikely to increase our understanding of this *M. xanthus* phenotype beyond a few hundred genes.

There are several ways we could increase the number of *M. xanthus* mutant strains that exhibit discernable phenotypic variation. We could increase the scope of environmental conditions; perhaps changing the temperature, light, salinity, osmotic pressure, pH or cell density would reveal more mutant strains with discernable phenotypic variation (North et al., 2012). We could develop new experimental methods to acquire data; perhaps a new fluorescence marker or microscopy method would uncover discernable traits that were previously overlooked. The problem is that, without a specific biological justification for selecting a new variable or method, there is no reason to believe that our ability to identify new mutant phenotypes would increase more than another few percent, if at all. Rather than developing anything experimentally novel, we hypothesized that we could identify new mutant phenotypes by simply recording and analyzing conventional data more rigorously and in greater detail.

To test this hypothesis on a representative subset of genes in the *M. xanthus* genome, we selected the ABC transporter family for the following reasons: (1) ABC transporters are diverse with respect to molecular function, and therefore are likely to impact a great variety of cellular systems; (2) ABC transporters are ubiquitous, and have been extensively studied in other organisms, so that meaningful differences in their putative molecular and cellular functions can be gleaned from an analysis of sequence homology and other organisms' annotations; (3) ABC transporters are not transcriptional regulators, thus discussions about molecular function do not invite the same degree of speculation regarding “direct” and “indirect” effects on the transcription of downstream genes; (4) the 192 ABC transporter ORFs we identified in *M. xanthus* represent a significant subset (2.6%) of the ORFs in the *M. xanthus* genome (Goldman et al., 2006), including 47% of the total number of transporters that are known to be directly involved in the molecular exchange between extra- and intra-cellular environments (Kuspa and Kaiser, 1989; Shi and Zusman, 1993; Kretzschmar et al., 2012).

ORFs encoding ABC transporters are abundant in bacterial genomes. They function in nutrient uptake, osmohomeostasis, signal transduction, and membrane synthesis. Prokaryotic ABC transporters include importers and exporters, both of which have four core domains: two ATPase domains and two transmembrane helix clusters (TMCs). Typically each subunit ORF contains only one core domain, although there are instances where one ORF codes for two or more domains. For importers there is usually a third subunit located in the periplasm (or anchored to the outer membrane in Gram-positive bacteria) called substrate binding protein (SBP). For this study, we targeted each subunit separately.

## Materials and methods

### Annotation of ABC transporters

The sequence of *M. xanthus* was obtained from GenBank (http://www.ncbi.nlm.nih.gov/GenBank/) with the accession number NC_008095.1. Each predicted ORF in the genome was annotated using multiple databases. ABC transporter-associated genes in the *M. xanthus* genome were reviewed and identified mostly using the databases pfam (Sonnhammer et al., 1997; Bateman et al., 2000; Finn et al., 2010) and COG (Tatusov et al., 1997). Additional tools such as BLAST (Altschul et al., 1990), InterPro (Apweiler et al., 2000; Mulder et al., 2002), GenBank (Benson et al., 2008) and transmembrane prediction server TMHMM Server v. 2.0 (Krogh et al., 2001) (http://www.cbs.dtu.dk/services/TMHMM/) were used to assist in the selection. A manual curation was adopted to complete the annotation. Briefly, an ORF was deemed an ABC transporter when at least two databases identified it as such. In order to ensure that all putative ABC transporter-associated ORFs were identified in the genome, a list of pfam accession IDs associated with these ORFs was compiled and used to search the rest of the genome. ORFs annotated as “hypothetical” that were located in the same operon with ABC transporter coding ORFs were manually checked using pfam and psi-blast. TMHMM was used to predict transmembrane domains in putative permeases. Using these methods, two additional ABC transporter-associated ORFs were identified, resulting in a total of 192 putative ABC transporter component coding ORFs.

### Cultivation and development

*M. xanthus* strains were grown at 32°C in CTTYE broth [1.0% Casitone, 0.5% yeast extract, 10 mM Tris-HCl (pH 8.0), 1 mM KH_2_PO_4_, and 8 mM MgSO_4_] or on plates containing CTTYE broth and 1.5% agar. CTTYE broth and plates were supplemented with 40 μg/ml kanamycin sulfate as needed. Cells underwent development on TPM agar [10 mM Tris-HCl (pH 8.0), 1 mM KH_2_PO_4_, 8 mM MgSO_4_, and 1.5% agar] at 32°C for 5 days.

### Mutagenesis

Primers for amplifying internal fragments of *M. xanthus* ORFs were selected using primer3 (http://sourceforge.net/projects/primer3/). The procedure for homologous recombination by plasmid insertion has been described previously (Caberoy et al., 2003). Briefly, an internal fragment of 400-600 bp was amplified using the polymerase chain reaction (PCR), and ligated into a linear plasmid pCR®2.1-TOPO (invitrogen). After ligation, the plasmid now includes the PCR product, and thus can be amplified in TOP10 *E. coli* cells. The plasmid was then isolated from *E. coli* and electroporated into *M. xanthus* cells (650 V, 25 μF, 400 Ω). The transformed plasmid was incorporated into the *M. xanthus* chromosome by homologous recombination (Plamann et al., 1994), thus conferring kanamycin resistance on the cells. In order to confirm that the plasmid was successfully inserted into the desired location in the *M. xanthus* chromosome, we used PCR to amplify across the upstream region of the target gene loci and TOPO vector, thereby generating an amplicon with size ~1.2 kb. Wild type DK1622 was used as negative control.

### Phenotype assays

*M. xanthus* cells were inoculated in CTTYE broth, cultivated with vigorous agitation (300 rpm) over night, and harvested at the density of ~5 × 10^8^ cells/ml. For motility assays, four spots of 2 μl cells at a concentration of 5 × 10^9^ cells/ml were placed on CTTYE plates containing 0.4 or 1.5% agar. Plates were incubated at 32°C for 3 days, and the diameters of colonies were then measured. For cell development, cells were washed once with TPM buffer and resuspended at a concentration of 5 × 10^9^ cells/ml in TPM buffer. Spots of 20 μl cell resuspension were spotted on TPM agar and incubated at 32°C for up to 5 days. The development of aggregates was observed and recorded at designated time intervals using 40× brightfield microscopy (Nikon) and SPOT imaging software. For the sporulation assay, cells were spotted on TPM agar and incubated at 32°C for 5 days to allow full development. Three sets of 5 spots were harvested and suspended in 500 μl TPM buffer. The cells were then exposed to mild sonication (10% altitude, 10 s × 3 with 30 s intervals, MISONIX, S-4000), followed by heat treatment at 50°C for 2 h. Cells were then diluted to the desired concentration and plated with CTTSA [1.0% Casitone, 10 mM Tris-HCl (pH 8.0), 1 mM KH_2_PO_4_, and 8 mM MgSO_4_, 0.7% agar] onto CTTYE agar plates (supplemented with 40 μg/ml kanamycin sulfate for insertion mutants). After 5 days of incubation at 32°C, viable spores germinated and grew into visible colonies, and the number of colonies was recorded and converted to the unit of cells/ml.

### Image analysis of development

At each time point of development on TPM agar, we examined at least five spots of 10^8^ cells. Brightfield images were taken for two of the five spots using a Nikon microscope and SPOT Insight camera (model #11.0 monochrome w/o IR) and imaging software at 40× magnification, and images were saved as non-reduced.tiff files. A.tiff file was selected because it preserves image quality and is lossless. To avoid the effect that the edge of the spot would have on image analysis, a section of each image representing 25% of the original that did not include the spot edge was submitted for analysis. ImageJ (http://rsbweb.nih.gov/ij/index.html) (Abramoff et al., 2004) was used to analyze the features of aggregates. These images were thresholded using the RenyiEntropy macro, and then manually corrected if necessary. After the area of each aggregate was selected for analysis, any aggregate that was less than 200 pixels (background noise) or that overlapped the edge of the image was excluded.

### Microarray analysis

The microarray data series GSE9477 for the ABC transporter project was retrieved from NCBI Gene Expression Omnibus (GEO) and normalized using SNOMAD (http://pevsnerlab.kennedykrieger.org/snomadinput.html, Copyrighted © 2000) (Colantuoni et al., 2002).

### Data analysis

We analyzed the extent of phenotypic variation in the mutant strains by comparing the mean ± *SD* across three independent replicates for each trait separately. For two of the traits we did not generate an *SD*; count was presented simply as the total number of aggregates observed, and timing, because it was recorded at five discrete intervals, was insufficient to resolve variation between replicates. To identify mutant phenotypes that are statistically distinct from wild type, we used a randomization test with 1000 iterations. This method generates *p*-values regardless of the distribution of the raw data, and we adjusted the *p*-values using the Benjamini, Hochberg, and Yekutieli method to control for any false discovery rate. To investigate whether phenotypic traits are correlated within mutant strains, we used a combination of Spearman's rank correlation (both zero-order and partial correlations), cluster analysis, and PCA. In the correlation analysis, we added the SDs from three phenotypic variables; in order to exclude the possibility that a large variation is caused by large average values (e.g., large average fruiting body area will also have a larger variation), we normalized the *SD* using std/mean. We used the Ward hierarchical clustering method to investigate how phenotypic traits were associated, and performed a separate clustering analysis on the mutant strains to look for overall similarities of phenotype. In the clustering and PCA analyses, data were converted to z-scores to preserve their distribution in variance while converting them to comparable units. Correlation analyses, multiple range tests, cluster analysis and PCA were all executed and visualized using R (Team, 2012) and MATLAB. Heatmaps were constructed using the R package amap (Lucas, 2011) and gplots (Warnes et al., 2011); bar plots were constructed using the R package gplots and colorspace; partial correlation was calculated with the package ppcor (Kim, 2012); box plots were constructed using R the package sfsmisc (Martin Maechler, 2012).

## Results

### Identification of ABC transporter ORFs in *M. xanthus*

**Figure 1:**
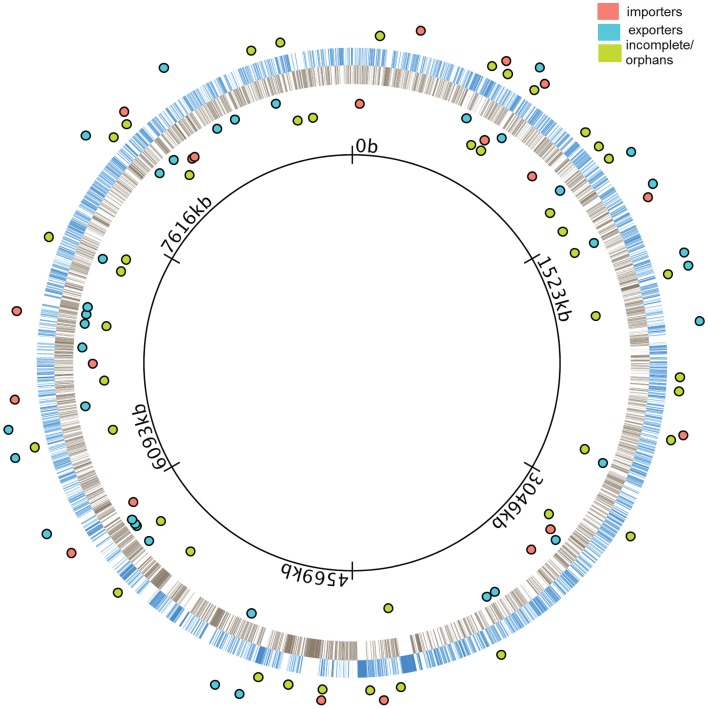
**Distribution of ABC transporters in the *M. xanthus* genome**. The innermost black ring represents the *M. xanthus* chromosome. Blue bars in the outer ring represent the genes transcribed in the clockwise direction (+ strand) while gray bars in the inner ring represents the genes transcribed in the counterclockwise direction (− strand). Colored dots represent operons containing coding genes for ABC transporters. The location of dots indicates that they are either in the + strand (outside blue ring) or in the − strand (inside black ring). Cyan and salmon colored dots represent full operons coding for complete exporters and importers, respectively. Green colored dots represent incomplete and orphan operons.

We performed a sequence-level characterization of all ABC transporters in the *M. xanthus* genome. To accomplish this, we carefully examined the entire genome using a combination of pfam (Bateman et al., 2000; Finn et al., 2010), COG (Tatusov et al., 1997, 2003), GO (Ashburner et al., 2000) and GenBank (Benson et al., 2008). The results are included in Figure 1. Once we had established the number, type, and distribution of ABC transporter component ORFs, we performed insertion-disruption mutagenesis on all 192 of them. Among these there were 12 that contain an ATPase domain and a TMC, and three that contain a TMC and an SBP. The rest contain only one of the three types of components, either an ATPase, a TMC, or an SBP. A total of 139 of the 192 ORFs were predicted to be coding for components of 57 complete ABC transporters, 20 importers and 37 exporters, based on our observation that they clustered within operons. The remaining 53 ORFs either form operons incomplete for an ABC transporter, or they are located alone in the genome.

### Mutagenesis results and phenotypic assays

We succeeded in creating 180 ABC transporter insertion-disruption mutant strains. For the remaining 12, we made three independent attempts, and each time no viable colonies were produced. We labeled all of these 12 ABC transporter ORFs “putative” essentials, since we did not perform a standard complementation assay to confirm that they were essential. It is important to note that this number is close to previous estimations of the percent of essential genes in *M. xanthus* (Kroos et al., 1986). For a list and brief description of the putative essential ORFs, please refer to Supplementary Material and Table S1.

**Figure 2:**
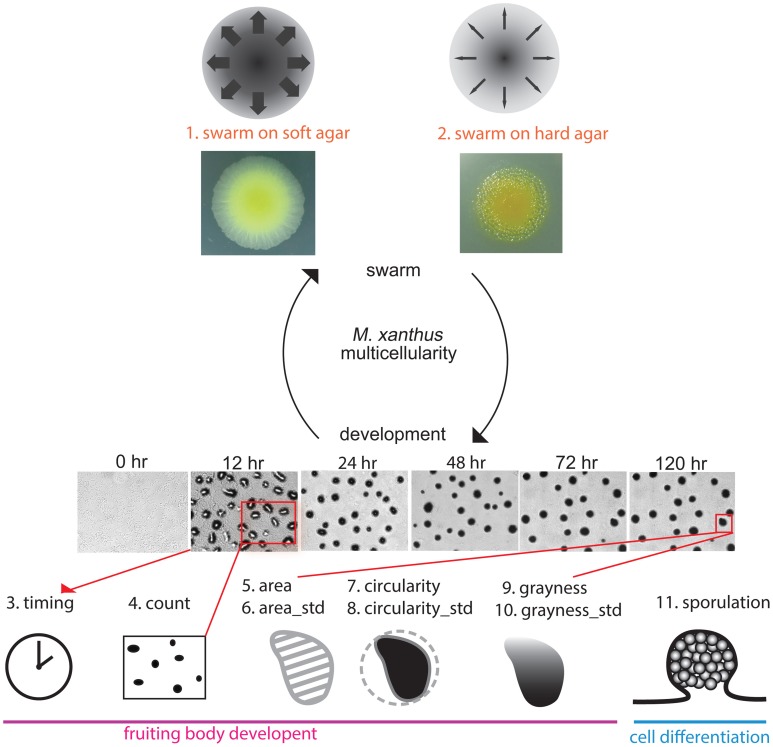
**Phenotypic traits and phenotypic assays**. We tested eight phenotypic traits spanning the two parts of the *M. xanthus* life cycle: swarming (top) and development (bottom). Two data sets related to swarming were obtained under rich media using either soft (0.4%) or hard (1.5%) agar as rough measurements of S and A motility, respectively. Images of the two yellow colonies are swarms after 3 days on both agar concentrations. Six data sets related to development were obtained under nutrient starvation. The six panels with times listed above are images of wild type development. Sporulation is also related to development, as well as cell differentiation. Drawings representing each phenotypic trait will be used to denote those traits in Figure **8**.

All of the assays we performed for this study used methods for measuring mutant phenotypes that are considered standard for *M. xanthus* laboratory research. Each assay focused on either swarming or development, and we examined a total of eight phenotypic traits: (1) and (2) the expansion rate of a swarm on both 0.4% (soft) and 1.5% (hard) agar surfaces, taken as a rough estimation of Social and Adventurous motility systems, respectively; (3) the time required for development (timing); (4) the opacity of aggregates as an indication of density (grayness); (5) the circularity of aggregates (circularity); (6) the number of aggregates in one unit area (count); (7) the average size of aggregates (area); and (8) the efficiency of sporulation (sporulation). We refer to each phenotypic trait using the above name in parentheses. For three of these variables (circularity, grayness, area), we quantified what is typically reported as a qualitative observation, but each one measures an aspect of the *M. xanthus* phenotype that is frequently described (Kuspa and Kaiser, 1989; Caberoy et al., 2003). Figure 2 illustrates how each assay is related to the *M. xanthus* phenotype and lifecycle.

### Distribution of phenotypic variation

**Figure 3:**
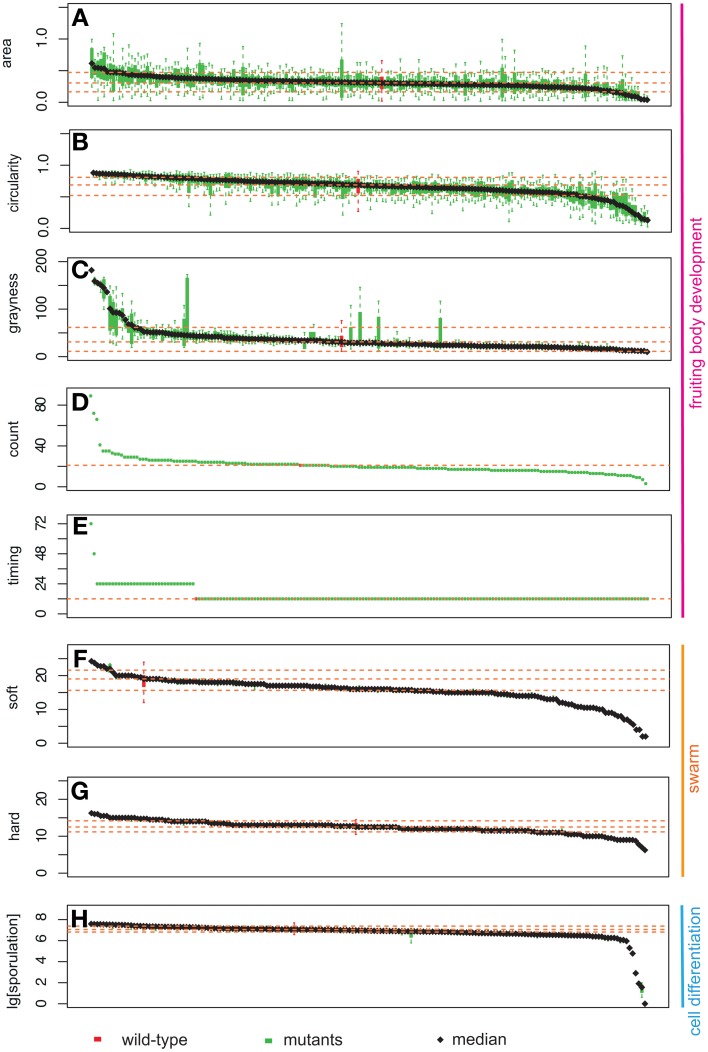
**Distribution of phenotypic data: area (A), circularity (B), grayness (C), count (D), timing (E), expansion on soft (F), and hard agar (G), and sporulation efficiency (H)**. The y-axis represents the measurement for each phenotypic assay. Bars on the x-axis represent 180 mutants and wild type. Green bars represent mutant strains, while red bars represent wild type. Box plots represent two middle quartiles. Error bars represent the top and bottom interquartile range for each strain. Yellow dash lines represent the mean ± *SD* (top, bottom), and median for wild type. In **(D,E)**, no error bars are present due to the nature of our measurements (for details, please see Materials and Methods).

For each of the phenotypic assays, data from wild type and the 180 mutant strains were listed in decreasing order according to their resultant means on the x-axes and the experimental values for each phenotypic trait on the y-axes (Figure 3). Therefore, the order of mutant strains is different for each graph in Figure 3. The range of phenotypic variation is different across the traits: e.g., sporulation ranges from 0 to 270% of wild type, soft and hard expansion are from 10 to 130% and 50 to 125% of wild type, respectively, and count is from 0 to 4-fold greater than wild type. Nonetheless, the distributions of all eight phenotypic traits in Figure 3 have at least four notable features in common: (1) all display a continuous distribution; (2) the majority of mutant strains fall within a confidence interval one standard deviation about the mean; (3) wild type is always within this confidence interval, usually near the middle; (4) mutant strains with “outlier” phenotypes (i.e., ones located where the slope sharply changes at either end) always represent a small percentage of the overall population.

To identify mutant phenotypes that are statistically distinguishable from wild type, we used a randomization test. Because the distributions of each phenotypic trait are continuous, it is difficult to establish meaningful thresholds that can be used to distinguish a set of strains as having one or more “mutant phenotypes.” We used a resampling strategy to compare each strain to wild type, and used an adjusted *p*-value to control for false discovery rates (see materials and methods). Using this method, we identified the number of mutant strains that are different from wild type for each phenotypic trait—area: 39, circularity: 91, grayness: 46, hard: 37, soft: 59, and sporulation: 62. A total of 86% of strains (154/180) exhibited at least one phenotypic trait that was statistically distinguishable from wild type. The results of the randomization test are listed in Table S1.

### Measuring pleiotropy among phenotypic traits

A total of 93 mutant strains (52%) exhibited some degree of pleiotropy, with at least two phenotypic traits statistically distinguishable from wild type; for example, strain MXAN_1097 exhibited both a slow rate of expansion on soft agar (soft) and reduced sporulation efficiency (sporulation). To characterize pleiotropic effects in *M. xanthus*, we examined correlations among phenotypic traits using Spearman's rank correlation coefficient for all 180 mutant strains. For several strains, we noticed a large variation in the area, grayness, and/or circularity of aggregates within the same swarm. We therefore analyzed means and standard deviations separately for these three traits.

**Figure 4:**
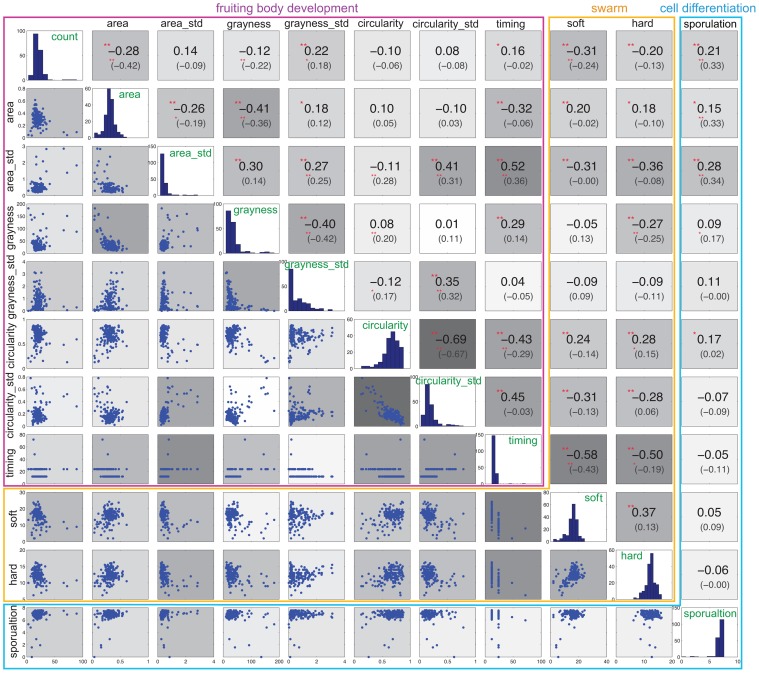
**Correlation of phenotypic variables**. The histogram in the diagonal panels shows the distribution of each phenotypic trait. The value of Spearman's rank correlation is the first number shown above the diagonal. The number inside the parenthesis is the Spearman's rank partial correlation. The background color above and below the diagonal corresponds to the degree of correlation, where deeper gray indicates a higher correlation. Significance correlations are indicated with asterisks: ^**^0.01, ^*^0.05.

Figure 4 shows the correlation between the now eleven traits (with area, circularity, and grayness reported as both average and standard deviation “_std”). Each trait exhibits several positive or negative correlations. Some support common sense hypotheses or confirm long-standing empirical observations. For example, soft and hard expansion exhibit a strong positive correlation with each other, and timing exhibits a strong negative correlation with both soft and hard expansion, thus indicating that slower swarm expansion is linked to slower development. Other correlations may seem less obvious; for example, sporulation exhibits no correlation with soft or hard expansion, or with most of the phenotypic traits associated with development, except for count and circularity.

In addition to this zero-order correlation, we also calculated the partial correlation coefficients between each pair of phenotypic traits, and found that they decreased in some cases (Figure 4, upper panels, inside parenthesis). In particular, five pairs of phenotypic traits associated with development exhibit decreased partial correlations: timing and count, timing and area, timing and grayness, timing and circularity_std, area and grayness_std. For each of the three phenotypic variables represented by both average and standard deviation (area, grayness and circularity), each average still exhibits a correlation with its corresponding standard deviation, and all three standard deviations are still correlated with each other. Sporulation correlates only with count, area, and area_std, but is no longer correlated with circularity.

Interestingly, the partial correlations associated with soft and hard expansion are diminished significantly. Neither soft nor hard expansion is correlated with area or area_std. More surprisingly, soft and hard expansion are no longer correlated with each other, which indicates that they are more independent with respect to phenotype than may have been previously assumed. Furthermore, soft and hard expansions now exhibit different correlations; count correlates only with soft, while grayness and circularity correlate only with hard.

To summarize, partial correlation analysis reveals that the phenotypic traits associated with development are more closely correlated with each other (11 of 28 with *p*-value < 0.05, or 36%) than with phenotypes associated with swarming (soft and hard expansion) (5 of 18 with *p*-value < 0.05, or 28%). Also, the correlation of other phenotypes with soft and hard expansion shows patterns different from their zero-order correlations, and they are also more different from each other, with soft exhibiting a correlation with only two of 10 phenotypic traits, namely timing and count.

### A comparison of wild type and mutant development

**Figure 5:**
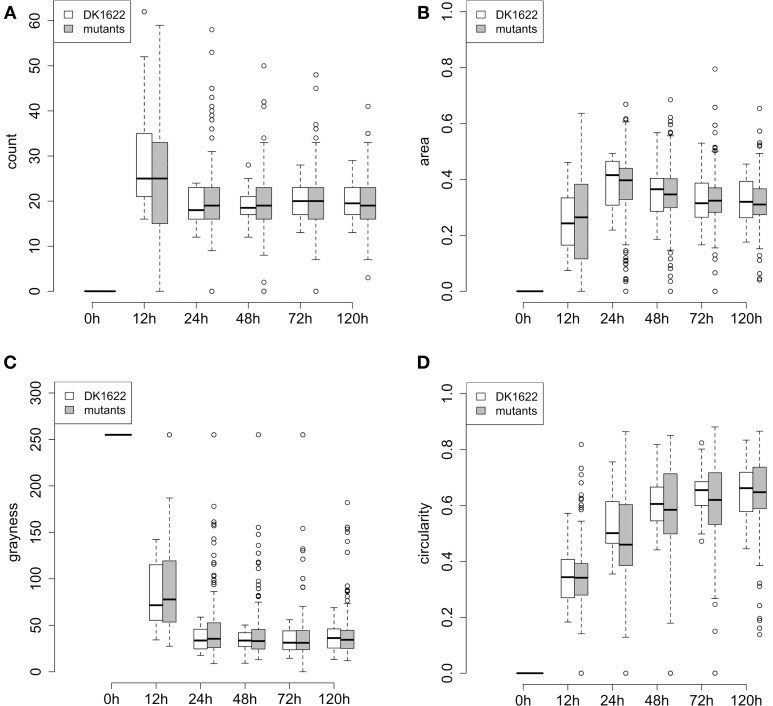
**Phenotypic traits of wild type and mutant aggregates during development (5 days)**. **(A–D)** Represent the quantitative traits for fruiting body development: **(A)** count, **(B)** area, **(C)** grayness, and **(D)** circularity. At each time point, the box represents the middle 50% of the data points, together with the median (thick line in each box) for the 180 mutants or DK1622 wild type replicates. Error bars represent the 1.5 interquartile ranges. Small circles above and below each error bar represent outliers.

A quantitative description of swarm patterns that form during development has been previously reported using time-lapse microcinematography images (Welch and Kaiser, 2001; Xie et al., 2011; Zhang et al., 2011), but these kinds of observations have not been reported for a collection of mutants. Here, we used the four phenotypic traits extracted from our images (count, area, grayness, and circularity) to describe the dynamics of development for the 180 ABC transporter mutant strains over 5 days. Changes in each of these four traits are plotted in Figure 5, and they are compared to 24 independent replicates of wild type. For the five time points of the four traits shown in Figure 5, the averages and standard deviations of all the mutant strains are actually very similar to the replicates of wild-type, although almost all of the outliers are mutant strains.

### Clustering of mutant strains based on phenotype

**Figure 6:**
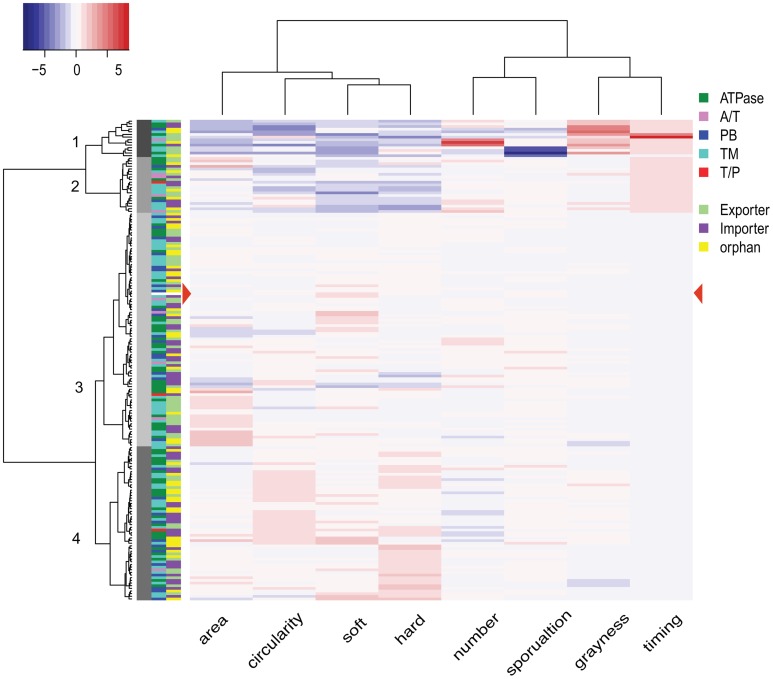
**Phenotype clustering of 180 mutant strains**. The dendrogram on the left represents the results from the clustering analysis of 180 mutant strains, with the numbers on each node indicating the four clusters. The dendrogram at the top is the result of hierarchical clustering of the eight phenotypic variables. The heat map key on top left shows the color indication for each z-score, with more intense shading indicating a greater deviation from wild type. Color filled tiles at the left of the heat map indicate two types of classification for each of the 180 mutant strains. The first classification is based on subunits (components of transporters), and the second classification is based on operon organization. A/T, ATPase/transmembrane permease fusion protein; PB, periplasmic binding protein; TM, transmembrane permease; T/P, transmembrane/periplasmic fusion protein.

We next considered the set of all phenotypic traits as a profile and clustered the strains accordingly (see Materials and Methods for a detailed description). The 181 mutant strains including wild type resolved into four groups, containing 14 (Group 1), 21 (Group 2), 88 (Group 3), and 58 (Group 4) strains, respectively (Figure 6, side dendrogram). We also performed a clustering analysis on the phenotypic traits. The eight traits resolved into two groups (Figure 6, top dendrogram). Group 1 includes both soft and hard expansion, as well as area and circularity. Group 2 includes the rest of the phenotypic traits associated with development, including sporulation.

Among the four groups of mutant strains, Group 1 is made up of 14 strains with phenotypic traits most distinguishable from wild type, with features including small area, low circularity, diminished soft and hard expansion, high count, low sporulation, increased grayness (i.e., lighter and more opaque), and delayed timing. These 14 Group 1 strains include mutations in five ATPases, seven transmembrane permeases (TMs), one substrate binding protein (SBPs), and one fused A/T (ATPase/TMs) protein. Group 2 is made up of 21 mutant strains that also display a range of phenotypic traits easily distinguishable from wild type although, when considered as a set, the mutant strains of Group 2 are less different from wild type than the mutant strains of Group 1. Group 2 strains display decreased circularity, reduced soft and hard expansion, and delayed timing; but sporulation efficiency, grayness and count are closer to wild type. Group 2 contains strains with mutations in six ATPases, eight TMs, two SBPs, four fused A/T proteins and one fused T/P (TMs/SBPs) protein.

Groups 3 and 4 include mutant strains with phenotypic traits that are more similar to wild type, and wild type is located within Group 3 (Figure 6, red arrowhead). When compared to Group 3, Group 4 is enriched for strains with increased circularity and increased soft and hard expansion, as well as a slightly lower count. Group 3 contains strains with mutations in 29 ATPases, 36 TMs, 18 SBPs, four fused A/T proteins, and one fused T/P protein. Group 4 contains strains with mutations in 22 ATPases, 23 TMs, 12 SPBs, one fused A/T protein, and one fused T/P protein. In other words, every type of ABC transporter component is represented in each of the four groups.

### Co-clustering of mutant strains based on expression and phenotype profiles

**Figure 7:**
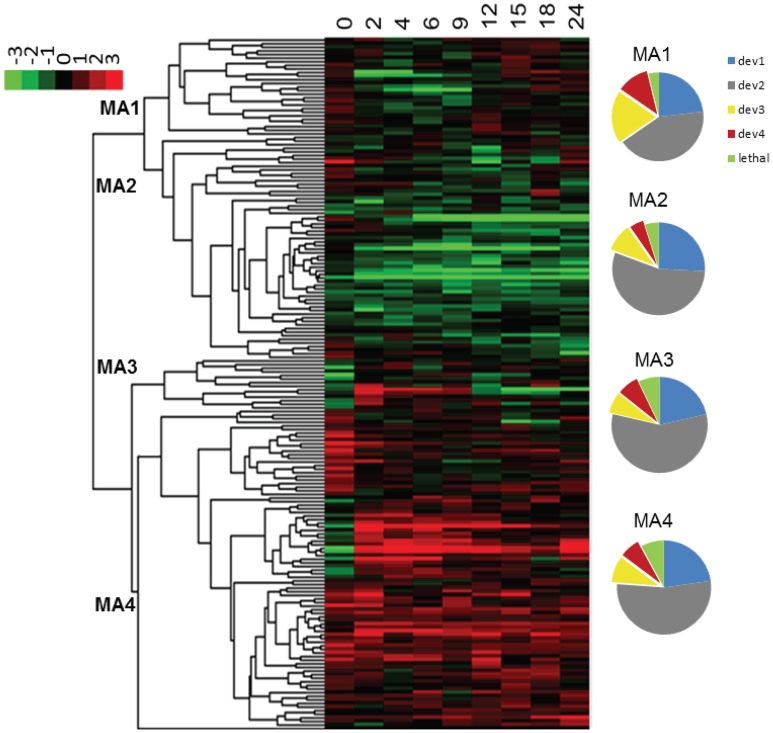
**Expression profiles of ABC transporters upon starvation**. The color scheme is shown in the upper left corner. The four clusters are numbered at their nodes. The numbers at the top of the heat map represent the hours after the initiation of starvation. The pie charts on the right show the composition of the four expression clusters (MA1-4). Different colors indicate the percentage of genes in each cluster (dev1–dev4, and putative lethal). MA, microarray cluster; le, potential lethal; dev, cluster by developmental phenotype.

Expression profiles sometimes provide indirect information regarding the function of a gene or the role it plays in shaping a phenotype. In order to examine the relation between phenotypes and expression profiles of ABC transporters during development, we examined microarray data from a wild type *M. xanthus* developmental time course that had been submitted to the NCBI GEO database (GSE9477). We first normalized the data using SNOMAD (Colantuoni et al., 2002), and then clustered them with Cluster 3.0 (Eisen et al., 1998) using the average linking method; the results are visualized in Figure 7. MXAN_5747 expression data were missing from the microarray report, but the other 179 ABC transporter subunit ORFs clustered into four major groups. ORFs in the first cluster (MA1) were mostly induced between the 12th and 24th hour after the initiation of starvation, whereas the ORFs from the second cluster (MA2) were mostly silent during development. The third cluster (MA3) contained mostly ORFs that were activated early (2nd–9th hour), and the fourth cluster (MA4) included ORFs that were nearly constitutively expressed.

It is assumed that bacteria express genes when they need them. If this is true for *M. xanthus*, then development expression profiles and phenotypic traits associated with development should be correlated. We therefore examined the six phenotypic traits related to development (count, area, grayness, circularity, timing, and sporulation), and compared the clustering similarity of these data with the microarray time course of development. Clustering analysis was again applied to the mutant strains, but with only the six phenotypic variables mentioned above (Figure S1). We observed that the 181 strains (mutants plus wild type) resolved into four clusters (dev1–dev4), and contain 45, 101, 21, and 14 strains respectively. When combined, dev3 and dev4 contain 35 mutant strains with development delayed by 12–48 h, and small irregularly shaped fruiting bodies. As expected, the strains in dev3/4 are the same ones that form groups 1 and 2 in the previous cluster analysis that included all eight phenotypic variables. A comparison between the dev1-4 clusters and the expression profiles in Figure 7 reveals that the 35 dev3/4 strains, which have the most distinguishable mutant phenotypes, appear disproportionately in cluster MA1, which represents ORFs activated between the 12th and 15th hour after starvation. Therefore, these data and analyses support the hypothesis that expression profiles can provide insight into other phenotypes. The relationship between expression and phenotype has been previously described in *M. xanthus*, but only for transcriptional regulators (Keseler and Kaiser, 1995; Gorski and Kaiser, 1998).

### Principle component analysis (PCA) of phenotypic traits

**Figure 8:**
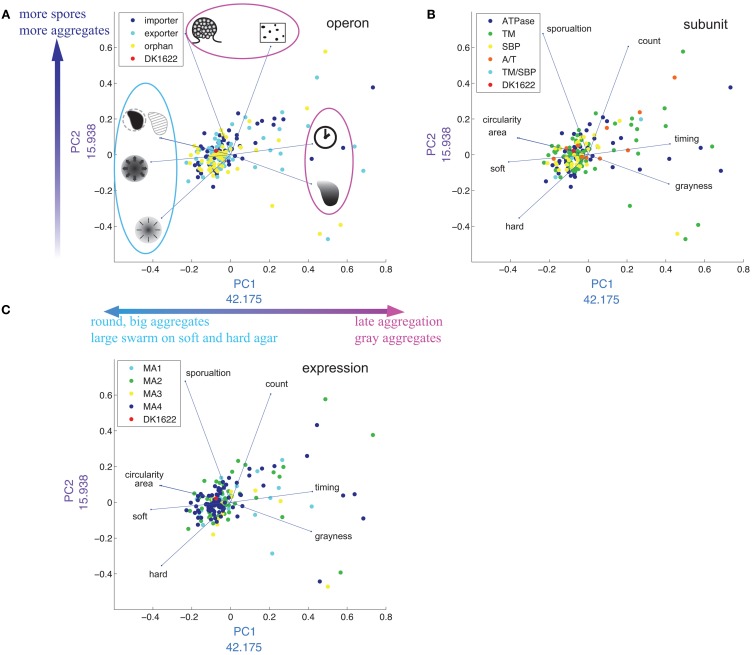
**Distribution of phenotypes in multidimensional scaling using PCA**. **(A–C)** Are the same plot of PC1 against PC2, except that the plots are color coded by operon **(A)**, subunit **(B)**, and expression cluster **(C)**. In **(A)**, drawings represent each phenotypic trait [see Figure 2, legend and corresponding text in plots **(B,C)**]. The numbers next to x- and y-axes indicate the weight of each PC in percent.

To reduce the complexity of separately considering eight or more *M. xanthus* phenotypic traits, we applied principal component analysis (PCA) to our data set (Figure 8). The first two principal components (PCs) account for more than 50% of the variance. PC1 is mostly a combination of six phenotypic traits: timing (positive), grayness (positive), soft and hard expansion (negative), area (negative) and circularity (negative). PC2 is a combination of three: sporulation (positive), count (positive) and hard expansion (negative). The PCA plot divides the eight phenotypic variables into three groups (Figure 8A), which is in good agreement with the cluster analysis (Figure 6: see dendrogram on top of heat map, groups I and II).

Timing and grayness have the highest loading values on PC1 (42.1% and 41.5%, respectively), indicating that they account for the largest differentiation among mutant strains, whereas sporulation and count have the highest loading values on PC2 (67.7 and 60.5%, respectively). As expected, the four phenotypic traits that load negatively with respect to PC1 (circularity, area, soft, and hard) are the same four that form group I in Figure 6. We also tested the hypothesis that PCA would reveal clustering associated with operon type (Figure 8A), subunit composition (Figure 8B), or expression patterns (Figure 8C), but in each case no clear pattern was observed.

The majority of mutant strains cluster around wild type (Figure 8), but the ones that do not are unevenly distributed. There are regions in the PCA graph that are entirely unpopulated.

## Discussion

From these data and analyses we have made several observations that apply to at least the 180 single gene disruption mutants in the *M. xanthus* ABC transporters. Our observations can be summarized in the following five statements: (1) By combining several quantitative measurements and applying randomization tests, 155 out of 180, or 86% of mutant strains were observed to exhibit at least one phenotypic trait that could be statistically distinguished from wild type. (2) The average wild type phenotypic trait closely follows the average for all 180 mutant strains, so that wild type was always within the confidence interval about the mean, and never represented an outlier for any phenotypic traits. (3) Phenotypic traits are not independent, so that observing one changed trait in a mutant strain alters the probability that other traits will also be changed, and this significantly impacts what should be considered an improbable phenotype. (4) None of the sequence-based data sets used in this study succeeded in co-clustering with phenotype, including sequence homology and annotation of molecular function, or genome topology and the prediction of ORFs. (5) Expression profiling data did co-cluster with the other phenotypes, indicating a correlation between the transcriptome and the phenome. For discussion of potential polar effects related to the generation of mutant strains using insertion disruption, please refer to Supplementary Material.

The impact of the first observation is the most obvious. The percent of strains that exhibit mutant phenotypes becomes higher when more than one phenotypic trait is included, the observation's comparison to wild type is quantitative, and setting the cutoff between what is wild type and what is mutant is not done completely arbitrarily, but instead is done using well known statistical procedures. By altering our experimental design and analysis accordingly, we were able to increase the percent of distinguishable mutant strains in *M. xanthus* to 86%, and this was using only a subset of standard *M. xanthus* phenotypic assays. These findings directly address the question of why previous studies have detected few *M. xanthus* mutant strains with distinguishable phenotypes: in part, it is because the limitations of our assays and analysis did not capture more subtle phenotypic changes.

The second observation impacts how we view each mutant strain's phenotypic traits and, by extension, the relationship between these standard laboratory assays and any real consideration of fitness. We disrupted 180 ORFs with no pre-selection for a specific phenotype, and found that between 3 and 26% of mutant strains performed “better” than wild type for any trait. This means that wild type is almost always somewhere toward the middle of the confidence interval about the mean, and this observation alone provides compelling evidence that fitness should not be defined through trait maximization, and assay results should be analyzed accordingly. Otherwise, by assigning wild type as “100%” of any phenotypic trait and then selecting only mutant strains that perform less than this arbitrary 100%, we will fail to distinguish a significant number of mutant phenotypes and inadvertently bias our results.

The impact of the third observation relates to the identification of outlier mutant phenotypes. If two phenotypic traits (A and B) exhibit a high degree of pleiotropy, so that for every mutant strain that exhibits a change in A there is always a change in B, then it is assumed that A and B must share some molecular underpinnings. In such a case, a strain that exhibits a large change in A with no change in B would be exceptional, and might be of particular interest to someone studying either trait. For example, *M. xanthus* aggregation and sporulation are correlated, so that mutant strains which fail to aggregate are much more likely to fail at sporulation. Therefore, a mutant strain such as MXAN_6671, that has been shown to sporulate without aggregating, represents a very interesting outlier mutant strain (Welch lab, unpublished data^*^).

(^*^The gene MXAN_6671 (sglK) has been previously disrupted using transposon insertion (Weimer et al., 1998; Yang et al., 1998) and was described as having defects in aggregation and a lower sporulation efficiency than wild type (5%). In our laboratory the disruption mutant displayed defects in aggregation and nearly wild type sporulation efficiency).

The fourth and fifth observations relate directly to our inability to accurately predict the behavior of any complex system; as the complexity of a system increases, our ability to predict the impact of small changes in the system decreases. This means that an analysis of sequence homology has a reasonably high success rate at predicting the effect of mutation on a protein's molecular function, a lower success rate at predicting its effect on cellular function, and an even lower success rate at predicting its effect on a multicellular organism's overall phenotype. This observation goes to the heart of why the genotype-to-phenotype problem seems intractable; it is comparable to accurately predicting the weather.

The impact of these observations also must be considered within the context of phenotypic profiling and the model organism. Most molecular model organisms were isolated from nature long ago, mutagenized and selected for tractability, and then studied for a few phenotypic traits that might be unrelated to fitness. Genome evolution has been redirected for these organisms, and it is not surprising that the phenotypic impact of mutation is difficult to detect under these conditions.

For a model organism such as *M. xanthus*, initial estimations of the dimensionality and scale of the phenome depend on the identification and characterization of outlier strains, since sets of phenotypic traits exhibited by these exceptional strains represent practical boundary values. Current definitions for the phenome of an organism are imprecise, but if the word “phenome” has any valid scientific meaning, it cannot be defined as infinite. Perhaps it would be logical to think of boundary values for a phenome as sets of phenotypic traits that are so unlikely to occur that their probability approaches zero. Therefore, by compiling sets of traits using standard assays for hundreds of *M. xanthus* mutant strains, we are just beginning to populate a map of the *M. xanthus* phenome. In this map, each independent phenotypic trait represents one full “dimension” of the phenome, two correlated traits represent more than one and less than two full dimensions, and a PCA represents something approaching the projection of a partial multidimensional phenome, with unpopulated regions indicating its practical boundaries.

### Conflict of interest statement

The authors declare that the research was conducted in the absence of any commercial or financial relationships that could be construed as a potential conflict of interest.
