# Supplementary material for: Phenotypic profiling of ABC transporter coding genes in Myxococcus xanthus
Source: Front Microbiol. 2014 Jul 18;5:352. doi: 10.3389/fmicb.2014.00352 (PMC4103005; doi:10.3389/fmicb.2014.00352)
Supplement: Supplementary file 1 [file DataSheet1.PDF]

1. Supplementary Material

This section may be divided by subheadings. This section should contain sufficient detail so that when read in conjunction with cited references, all procedures can be repeated.

1.1. Potential lethal mutations

This group includes 12 ORFs, whose length ranges from 675 bp to 2730 bp. We made at least three independent attempts to produce an insertion-disruption mutant strain for each of these ORFs, but no viable colonies were obtained. For four of them (MXAN_4730, MXAN_4788, MXAN_4789, MXAN_4791), we tried four different pairs of primers. Based on this evidence, we hypothesize that mutations in those 12 loci are lethal, but refer to them as putative essentials because we did not confirm lethality through complementation. Without this confirmation, it is impossible to conclude that these genes are essential, because a less likely but feasible alternative hypothesis is that these 12 ORFs are difficult to disrupt because of complex secondary structure, or protein binding that makes them more resistant to homologous recombination. Some information regarding these 12 ABC transporters can be deduced from their sequence and neighboring genes.

The 12 ORFs are located in the genome as one orphan and seven complete operons. Four genes out of the 12 encode components of the only importer for inorganic phosphate (MXAN_4788 - MXAN_4791). This ABC transporter is located downstream of the phoP4 ORF, which encodes the response regulator of a two component system (Pham et al., 2006). The expression of MXAN_4788 and MXAN_4791 are highly elevated 48 h and 60 h after the initiation of starvation (Pham et al., 2006). There are three potential lethal ORFs that code for two fully functional exporters. MXAN_0748 is a fused protein with both an ATPase and a transmembrane cluster (TMC) as a half-transporter. Although surrounded by several hypothetical proteins, three downstream genes, a serine/threonine protein kinase Pkn9 (MXAN_0755), a response regulator (MXAN_0763), and an M24 family peptidase (MXAN_0764), hint that this ABC transporter may function in signal transduction. Another ABC transporter, formed by MXAN_4729 and MXAN_4730, is close to the LolC/E family lipoprotein releasing system, the transmembrane protein (MXAN_4730), and the OmpH family outer membrane protein chaperon (MXAN_4727), which makes it possible that this transporter functions in protein secretion.

The only orphan potential lethal (MXAN_1060, ATPase) is located next to an RNA polymerase sigma-54 factor (MXAN_1061), as well as genes related to lipopolysaccharide synthesis (MXAN_1052: O-antigen polymerase, MXAN_1057: lipid A biosynthesis acyltransferase, MXAN_1062: acetyltransferase, MXAN_1063: lipoprotein). This ABC transporter may be involved in outer membrane biosynthesis, although no functional partner has been identified.

There are also four potential lethal ORFs that encode subunits within complete operons, but none of them are the first ORF in the predicted operon. All four belong to predicted exporters. MXAN_1789 codes for a half-transporter, as does its potential partner MXAN_1790. They are located in the same operon with two other ORFs, one an HlyD family secretion protein (MXAN_1788), and the other an outer membrane efflux protein (MXAN_1791), which indicates that this ABC transporter could function in protein secretion. For the remaining three potential lethal ORFs, the ATPase MXAN_5699 is located close to one sensor protein kinase (MXAN_5704) and one serine/threonine protein kinase (MXAN_5696). The permease MXAN_6403, with its ATPase MXAN_6402, is

located close to a fatty acid synthesis cluster (MXAN_6392 - MXAN_6401). The ATPase MXAN_7145, with another ATPase MXAN_7144 and a transmembrane permease MXAN_7146, is located close to a sensory box histidine kinase (MXAN_7142) and a Fis family transcriptional regulator (MXAN_7143). The Fis protein in *E. coli* has been found to activate the antibiotic resistance operon marRAB (Martin and Rosner, 1997). Insertion in either one of the flanking genes results in an extreme phenotypic defect. Taken together, these three ABC transporters, along with MXAN_5699, MXAN_6403 and MXAN_7145, may be involved in signal transduction, secondary metabolite secretion and antibiotic resistance, respectively.

1.2. Potential polar effects

Although it is not the principle focus of this study, these data also provide some insight into one possible unintended consequence of insertion-disruption mutagenesis in prokaryotes that is commonly referred to as "polar effects". A polar effect occurs when the disruption of one gene in an operon alters the expression of downstream genes by early transcription or translation termination. Polar effects have been identified and studied in other organisms; one of the first was in the *trp* operon of *E. coli*, where expression of *trpB* was affected by mutation in the upstream *trp* genes (Jackson and Yanofsky, 1972). There has never been a published study that measured the frequency or extent of polar effects in *M. xanthus*. Therefore, our data provide some novel insight regarding *M. xanthus* polar effects, if one reasonable assumption is made.

Consider an operon consisting of three ORFs (A, B, and C) that are transcribed together, and each is translated into a protein product. If each of these three ORFs is disrupted independently through homologous recombination, the predicted impact of polar effects would be the following: disrupting C would eliminate functional C protein, disrupting B would eliminate functional B and C proteins, and disrupting A would eliminate functional A, B, and C proteins. This is the assumption behind the idea of polar effects and, if it is true, then the following three scenarios can be predicted: if eliminating C functional protein has a severe phenotypic impact, but eliminating A or B does not, then a polar effect would mean that disrupting ORFs A, B, or C would all produce strains that exhibit a severe phenotypic impact; if eliminating functional protein B has a severe phenotypic impact, but eliminating A or C does not, then a polar effect would mean that disrupting ORFs A or B would produce a strain with a severe phenotypic impact, whereas disrupting C would not; if eliminating functional protein A produces a severe phenotypic impact, but eliminating B or C does not, then polar effects do not apply, and disrupting ORF A would produce a strain with a severe phenotypic impact, whereas disrupting B or C would not.

In our study there are 34 mutants displaying very severe phenotypes, along with 12 that are potential lethals, and these 46 are in 32 predicted operons (Taboada et al., 2010). Among these, five are the first gene in the predicted operon, 24 are in the middle, 10 are the last, and seven are not in a multi-gene operon. Among the 10 that are last, there are three whose mutation displays the most severe mutant phenotype among all the disrupted ORFs in that operon. Relating these data to the discussion of polar effects in the previous paragraph, this means that in 3/10 (30%) of cases where, in an operon consisting of ORFs ABC, the disruption of ORF C had a severe phenotypic impact, whereas the disruption of ORFs A or B did not. Although the indirect nature of these observations and the small sample size means that we cannot accurately quantify the impact of polar effects in *M. xanthus*, these data do show that polar effects do not always occur when measuring mutant phenotypes. Any alternative explanation for these results that retains the impact of polar effects would also require additional assumptions about a disruption's effect on feedback and/or other hypothetical regulatory effects within the ORF, but these would also support our conclusion that polar effects do not have a

universal effect on phenotype.

2. Supplementary References

Jackson, E.N., and Yanofsky, C. (1972). Internal deletions in the tryptophan operon of *Escherichia coli*. *J Mol Biol* 71**,** 149-161.

Martin, R.G., and Rosner, J.L. (1997). Fis, an accessorial factor for transcriptional activation of the mar (multiple antibiotic resistance) promoter of *Escherichia coli* in the presence of the activator MarA, SoxS, or Rob. *J Bacteriol* 179**,** 7410-7419.

Pham, V.D., Shebelut, C.W., Jose, I.R., Hodgson, D.A., Whitworth, D.E., and Singer, M. (2006). The response regulator PhoP4 is required for late developmental events in *Myxococcus xanthus*. *Microbiology* 152**,** 1609-1620. doi: 10.1099/mic.0.28820-0.

Taboada, B., Verde, C., and Merino, E. (2010). High accuracy operon prediction method based on STRING database scores. *Nucleic Acids Res* 38**,** e130. doi: 10.1093/nar/gkq254.

3. Supplementary Figures

Table S1 Adjusted p-values from the randomization test, comparing each mutant strain to wild type (number of iteration = 1000).

MXAN#	hard	soft	area	circularity	grayness	sporulation
MXAN_0035	0.051	0.000	0.000	0.000	0.069	0.437
MXAN_0036	0.017	0.405	0.851	0.238	0.700	0.997
MXAN_0037	0.006	0.000	0.000	0.000	0.000	0.725
MXAN_0107	0.000	0.000	0.043	0.000	0.028	0.893
MXAN_0108	0.061	0.000	0.000	0.000	0.000	0.880
MXAN_0249	0.543	0.833	0.710	0.257	0.928	0.791
MXAN_0250	0.115	0.000	0.098	0.000	0.066	0.880
MXAN_0251	0.035	0.050	0.008	0.182	0.559	0.773
MXAN_0553	0.279	0.590	0.414	0.082	0.436	0.081
MXAN_0554	0.344	0.590	0.026	0.000	0.700	0.715
MXAN_0559	0.241	0.000	0.745	0.822	0.073	0.450
MXAN_0596	0.540	0.156	0.503	0.495	0.100	0.287

MXAN_0597	0.032	0.873	0.865	0.003	0.700	0.037
MXAN_0622	0.006	0.000	0.501	0.000	0.075	0.579
MXAN_0629	0.825	0.873	0.088	0.156	0.839	0.579
MXAN_0684	0.000	0.000	0.000	0.000	0.000	0.299
MXAN_0685	0.059	0.000	0.026	0.077	0.000	0.287
MXAN_0686	0.000	0.000	0.030	0.000	0.075	0.053
MXAN_0687	0.601	0.000	0.698	0.055	0.428	0.880
MXAN_0696	0.592	0.000	0.000	0.000	0.139	0.266
MXAN_0721	0.827	0.044	0.852	0.000	0.075	0.000
MXAN_0722	0.125	0.873	0.999	0.000	0.291	0.348
MXAN_0751	0.877	0.028	0.611	0.016	0.069	0.705
MXAN_0770	0.912	0.012	0.603	0.178	0.025	0.579
MXAN_0771	0.795	0.599	0.333	0.000	0.314	0.496
MXAN_0772	0.115	0.130	0.474	0.000	0.862	0.324
MXAN_0966	0.953	0.159	0.720	0.387	0.480	0.008
MXAN_0967	0.540	0.135	0.124	0.018	0.928	0.405
MXAN_0968	0.233	0.590	0.438	0.000	0.436	0.804
MXAN_0995	0.795	0.427	0.374	0.613	0.032	0.231
MXAN_1097	0.006	0.000	0.000	0.000	0.000	0.027
MXAN_1124	0.592	0.112	0.000	0.005	0.064	0.425
MXAN_1151	0.592	0.009	0.142	0.000	0.091	0.144
MXAN_1153	0.592	0.052	0.999	0.100	0.132	0.000
MXAN_1154	0.825	0.042	0.072	0.269	0.184	0.575
MXAN_1155	0.283	0.493	0.008	0.000	0.122	0.386
MXAN_1262	0.277	0.211	0.142	0.303	0.064	0.000

MXAN_1286	0.006	0.000	0.745	0.000	0.069	0.085
MXAN_1319	0.592	0.009	0.014	0.024	0.000	0.723
MXAN_1320	0.022	0.049	0.904	0.445	0.041	0.110
MXAN_1321	0.000	0.060	0.777	0.303	0.436	0.000
MXAN_1376	0.370	0.446	0.106	0.498	0.041	0.000
MXAN_1377	0.116	0.000	0.008	0.182	0.731	0.063
MXAN_1547	0.135	0.005	0.881	0.263	0.016	0.876
MXAN_1548	0.243	0.036	0.447	0.000	0.291	0.011
MXAN_1597	0.277	0.022	0.159	0.048	0.839	0.880
MXAN_1598	0.342	0.914	0.195	0.104	0.088	0.880
MXAN_1604	0.795	0.405	0.000	1.000	0.121	0.858
MXAN_1605	0.540	0.082	0.362	0.000	0.645	0.000
MXAN_1695	0.592	0.386	0.732	0.388	0.852	0.039
MXAN_1790	0.592	0.071	0.081	0.003	0.668	0.000
MXAN_2018	0.795	0.135	0.892	0.546	0.221	0.000
MXAN_2019	0.592	0.009	0.159	0.063	0.055	0.073
MXAN_2020	0.795	0.000	0.865	0.000	0.950	0.000
MXAN_2078	0.834	0.083	0.178	0.000	0.006	0.676
MXAN_2249	0.956	0.873	0.487	0.180	0.839	0.844
MXAN_2250	0.142	0.012	0.598	0.000	1.000	0.000
MXAN_2251	0.006	0.873	0.598	0.222	0.441	0.715
MXAN_2268	0.061	0.057	0.859	0.314	0.054	0.951
MXAN_2407	0.079	0.158	0.063	0.000	0.174	0.223
MXAN_2428	0.336	0.412	0.340	0.000	0.950	0.676
MXAN_2429	0.088	0.000	0.603	0.482	0.223	0.880

MXAN_2430	0.346	0.791	0.081	0.060	0.436	0.044
MXAN_2654	0.000	0.000	0.890	0.860	0.071	0.000
MXAN_2783	0.795	0.056	0.904	0.061	0.040	0.000
MXAN_2795	0.795	0.175	0.000	0.008	0.223	0.081
MXAN_2831	0.000	0.667	0.865	0.000	0.088	0.000
MXAN_2832	0.694	0.873	0.146	0.028	0.021	0.575
MXAN_2833	0.887	0.676	0.727	0.000	0.881	0.205
MXAN_2853	0.795	0.349	0.504	0.024	0.223	0.880
MXAN_2948	0.795	0.699	0.438	0.406	0.737	0.876
MXAN_2949	0.039	0.000	0.000	0.000	0.000	0.000
MXAN_2951	0.784	0.699	0.288	0.000	0.788	0.231
MXAN_3208	0.529	0.841	0.397	0.000	0.700	0.739
MXAN_3209	0.277	0.446	0.957	0.064	0.924	0.997
MXAN_3256	0.232	0.000	0.019	0.077	0.075	0.000
MXAN_3257	0.611	0.000	0.000	0.000	0.000	0.000
MXAN_3258	1.000	0.000	0.000	0.000	0.000	0.000
MXAN_3339	0.795	0.785	0.904	0.005	0.122	0.804
MXAN_3648	0.611	0.873	0.072	0.000	0.543	0.032
MXAN_3650	0.843	0.060	0.892	0.180	0.578	0.496
MXAN_3717	0.000	0.000	0.138	0.000	0.679	0.004
MXAN_3718	0.000	0.000	0.000	0.005	0.006	0.997
MXAN_3719	0.000	0.000	0.175	0.330	0.101	0.444
MXAN_3773	0.795	0.785	0.053	0.005	0.186	0.425
MXAN_3908	0.795	0.274	0.603	0.003	0.055	0.004
MXAN_3909	0.795	0.009	0.362	0.326	0.691	0.000

MXAN_3910	0.912	0.414	0.603	0.014	0.041	0.389
MXAN_3911	0.006	0.696	0.165	0.072	0.000	0.257
MXAN_3912	0.795	0.009	0.797	0.652	0.006	0.469
MXAN_3986	0.953	0.390	0.384	0.005	0.436	0.725
MXAN_4074	0.540	0.060	0.362	0.180	0.233	0.997
MXAN_4102	0.000	0.966	0.039	0.065	0.000	0.000
MXAN_4103	0.233	0.543	0.603	0.003	0.025	0.569
MXAN_4172	0.795	0.357	0.778	0.706	0.950	0.144
MXAN_4173	0.795	0.135	0.030	0.481	0.121	0.027
MXAN_4174	0.540	0.711	0.384	0.003	0.700	0.880
MXAN_4199	0.912	0.563	0.727	0.078	0.291	0.997
MXAN_4201	0.795	0.083	0.745	0.814	0.328	0.000
MXAN_4523	0.000	0.047	0.098	0.388	0.048	0.067
MXAN_4586	0.696	0.217	0.097	0.048	0.082	0.583
MXAN_4622	0.054	0.000	0.008	0.030	0.016	0.000
MXAN_4623	0.000	0.000	0.146	0.000	0.000	0.000
MXAN_4664	0.592	0.012	0.333	0.014	0.170	0.000
MXAN_4665	1.000	0.319	0.904	0.888	0.157	0.575
MXAN_4716	0.279	0.000	0.995	0.508	0.679	0.369
MXAN_4749	0.540	0.576	0.014	0.203	0.075	0.389
MXAN_4750	0.425	0.390	0.785	0.003	0.645	0.094
MXAN_4818	0.834	0.194	0.008	0.674	0.715	0.425
MXAN_4819	0.795	0.036	0.603	0.104	0.355	0.579
MXAN_4820	0.265	0.873	0.008	0.046	0.170	0.725
MXAN_4821	0.592	0.935	0.699	0.183	0.366	0.291

MXAN_4878	0.953	0.083	0.959	0.476	0.510	0.021
MXAN_4879	0.953	0.873	0.441	0.304	0.016	0.000
MXAN_5167	0.000	0.182	0.633	0.000	0.000	0.053
MXAN_5168	0.000	0.000	0.865	0.000	0.000	0.465
MXAN_5183	0.051	0.797	0.563	0.000	1.000	0.201
MXAN_5275	0.060	0.880	0.358	0.528	0.121	0.055
MXAN_5276	0.256	0.018	0.999	0.266	0.578	0.227
MXAN_5316	0.000	0.000	0.800	0.000	0.102	0.629
MXAN_5317	0.696	0.185	0.023	0.010	0.950	0.000
MXAN_5377	0.105	0.102	0.098	0.012	0.086	0.000
MXAN_5378	0.795	0.115	0.165	0.000	0.146	0.144
MXAN_5379	0.706	0.385	0.851	0.044	0.614	0.256
MXAN_5419	0.795	0.089	0.230	0.094	0.700	0.579
MXAN_5502	0.177	0.841	0.123	0.000	0.028	0.731
MXAN_5503	0.000	0.000	0.180	0.000	0.016	0.032
MXAN_5583	0.592	0.255	0.000	0.048	0.006	0.011
MXAN_5584	0.795	0.009	0.083	0.266	0.069	0.102
MXAN_5698	0.592	0.112	0.865	0.814	0.127	0.176
MXAN_5702	0.097	0.784	0.384	0.083	0.473	0.880
MXAN_5711	0.000	0.154	0.804	0.012	0.075	0.858
MXAN_5712	0.540	0.159	0.023	0.294	0.016	0.731
MXAN_5713	0.243	0.417	0.232	0.786	0.195	0.997
MXAN_5714	0.953	0.873	0.955	0.558	0.151	0.121
MXAN_5747	0.000	0.000	0.920	0.000	0.000	0.893
MXAN_5748	0.139	1.000	0.904	0.822	0.684	0.141

MXAN_5780	0.057	0.000	0.019	0.000	0.417	0.186
MXAN_5781	0.006	0.000	0.501	0.000	0.000	0.004
MXAN_5978	0.117	0.409	0.081	0.000	0.928	0.644
MXAN_6000	0.006	0.427	0.333	0.238	0.000	0.004
MXAN_6001	0.017	0.070	0.603	0.077	0.637	0.613
MXAN_6002	0.912	0.228	0.863	0.203	0.221	0.041
MXAN_6003	0.912	0.116	0.165	0.476	0.310	0.000
MXAN_6042	0.953	0.836	0.887	0.003	0.670	0.008
MXAN_6402	0.084	0.127	0.710	0.343	0.075	0.021
MXAN_6456	0.277	0.152	0.865	0.698	0.310	0.018
MXAN_6474	0.795	0.228	0.873	0.005	0.366	0.000
MXAN_6475	0.885	0.067	0.865	0.232	0.195	0.000
MXAN_6518	0.035	0.067	0.257	0.178	0.075	0.804
MXAN_6551	0.241	0.798	0.603	0.650	0.223	0.880
MXAN_6552	0.256	0.064	0.553	0.516	0.355	0.018
MXAN_6553	0.592	0.580	0.999	0.312	0.700	0.000
MXAN_6554	0.243	0.632	0.543	0.014	0.852	0.340
MXAN_6568	0.060	0.637	0.137	0.000	0.040	0.705
MXAN_6569	0.143	0.018	0.060	0.000	0.000	0.000
MXAN_6575	0.000	0.005	0.026	0.000	0.000	0.000
MXAN_6576	0.592	0.417	0.008	0.000	0.000	0.000
MXAN_6643	0.118	0.740	0.634	0.018	0.166	0.827
MXAN_6644	0.000	0.057	0.745	0.312	0.700	0.034
MXAN_6645	0.877	0.508	0.999	0.185	0.006	0.000
MXAN_6661	0.241	0.256	0.557	0.000	0.187	0.004

MXAN_6662	0.795	0.154	0.552	0.203	0.170	0.883
MXAN_6663	0.592	0.050	0.362	0.104	0.578	0.340
MXAN_6664	0.088	0.417	0.046	0.245	0.051	0.008
MXAN_6665	0.795	0.070	0.736	0.786	0.087	0.000
MXAN_6765	0.283	0.034	0.000	0.000	0.859	0.069
MXAN_6766	0.283	0.151	0.008	0.049	0.170	0.579
MXAN_6826	0.290	0.012	0.078	0.498	0.501	0.880
MXAN_6827	0.183	0.034	0.501	0.012	0.700	0.124
MXAN_6934	0.265	0.403	0.778	0.008	0.543	0.000
MXAN_7114	0.795	0.878	0.797	0.880	0.901	0.880
MXAN_7115	1.000	0.099	0.634	0.000	0.954	0.389
MXAN_7144	0.694	0.054	0.019	0.147	0.309	0.956
MXAN_7146	0.017	0.914	0.053	0.312	0.069	0.221
MXAN_7225	0.059	0.439	0.523	0.000	0.006	0.000
MXAN_7226	0.795	0.018	0.809	0.000	0.604	0.004
MXAN_7293	0.795	0.123	0.907	0.814	0.006	0.034
MXAN_7294	0.012	0.067	0.159	0.314	0.000	0.151
MXAN_7295	0.912	0.255	0.000	0.698	0.170	0.997

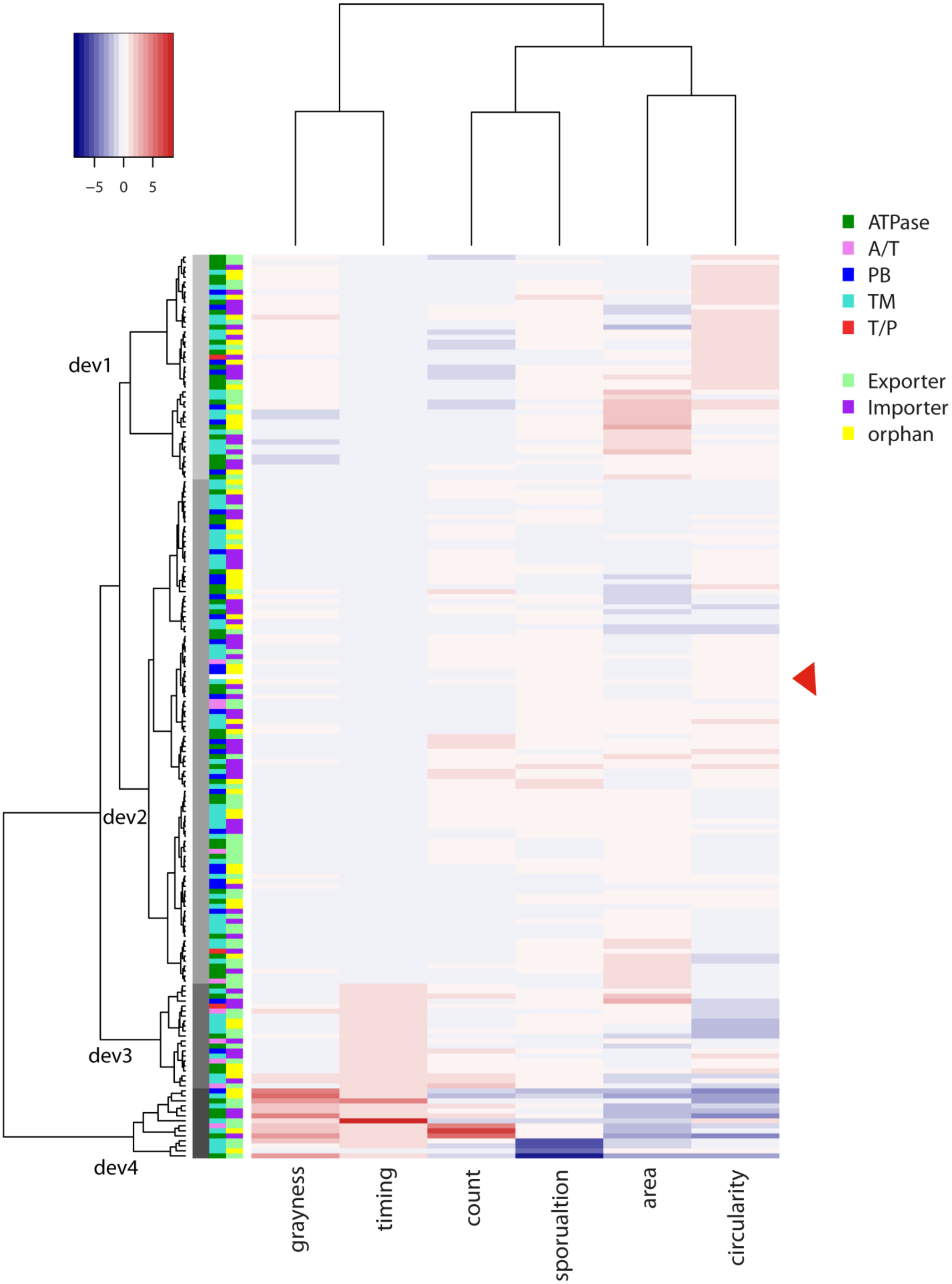

Figure S1 Cluster analysis of developmental phenotypes for the 180 mutants together with wild type (indicated by the red arrow head). The 181 strains resolve into four clusters dev1-dev4. The

dev4 cluster contains the mutants with the most distinguishable phenotypes. These four clusters are analyzed in figure 7 together with microarray data.
